# S4S8-RPA phosphorylation as an indicator of cancer progression in oral squamous cell carcinomas

**DOI:** 10.18632/oncotarget.14001

**Published:** 2016-12-16

**Authors:** Jeff Rector, Sasha Kapil, Kelly J Treude, Phyllis Kumm, Jason G. Glanzer, Brendan M. Byrne, Shengqin Liu, Lynette M Smith, Dominick J DiMaio, Peter Giannini, Russell B Smith, Greg G. Oakley

**Affiliations:** ^1^ Department of Oral Biology, College of Dentistry, University of Nebraska Medical Center, Lincoln NE 68583, USA; ^2^ Department of Otolaryngology/Head and Neck Surgery, University of Nebraska Medical Center, Omaha NE 68198, USA; ^3^ Department of Biostatistics, College of Public Health, University of Nebraska Medical Center, Omaha NE 68198, USA; ^4^ Department of Pathology and Microbiology, College of Medicine, University of Nebraska Medical Center, Omaha NE 68198, USA; ^5^ Eppley Institute for Research in Cancer and Allied Diseases, University of Nebraska Medical Center, Omaha NE 68198, USA

**Keywords:** replication protein A, squamous cell carcinoma, phosphorylation, tumorigenicity, immunohistochemistry

## Abstract

Oral cancers are easily accessible compared to many other cancers. Nevertheless, oral cancer is often diagnosed late, resulting in a poor prognosis. Most oral cancers are squamous cell carcinomas that predominantly develop from cell hyperplasias and dysplasias. DNA damage is induced in these tissues directly or indirectly in response to oncogene-induced deregulation of cellular proliferation. Consequently, a DNA Damage response (DDR) and a cell cycle checkpoint is activated. As dysplasia transitions to cancer, proteins involved in DNA damage and checkpoint signaling are mutated or silenced decreasing cell death while increasing genomic instability and allowing continued tumor progression. Hyperphosphorylation of Replication Protein A (RPA), including phosphorylation of Ser4 and Ser8 of RPA2, is a well-known indicator of DNA damage and checkpoint activation. In this study, we utilize S4S8-RPA phosphorylation as a marker for cancer development and progression in oral squamous cell carcinomas (OSCC). S4S8-RPA phosphorylation was observed to be low in normal cells, high in dysplasias, moderate in early grade tumors, and low in late stage tumors, essentially supporting the model of the DDR as an early barrier to tumorigenesis in certain types of cancers. In contrast, overall RPA expression was not correlative to DDR activation or tumor progression. Utilizing S4S8-RPA phosphorylation to indicate competent DDR activation in the future may have clinical significance in OSCC treatment decisions, by predicting the susceptibility of cancer cells to first-line platinum-based therapies for locally advanced, metastatic and recurrent OSCC.

## INTRODUCTION

Oral cancer is the sixth most common cancer with a poor five year survival rate, less than 50%. Genetic alterations are common in OSCC, including inactivation of tumor suppressors such as p53 and activation of oncogenes, including H-ras and cyclin E [1]. Overexpression of H-ras promotes overexpression of cyclin E [2]. Consequently, cyclin E increases origin firing resulting in replication stress and the activation of the DDR [3, 4]. The DDR is a complex network of proteins that work together to detect DNA damage and affect a cellular response through an extensively interconnected series of signal transduction pathways. Ultimately, DDR activation concludes with one of four outcomes: (1 Halting progression of the cell cycle, before resuming the cell cycle after repair of DNA damage, termed checkpoint activation and checkpoint recovery, respectively; (2 Apoptosis – programmed cell death; (3 Senescence – permanent cell cycle arrest; or (4 Bypassing the DDR and resumption of the cell cycle without complete DNA repair, leading to genetic instability, and in some cases, uncontrolled cell proliferation and cancer formation [5].

It is clear that there are enormous biological consequences of a mutated or incapacitated DDR. Through better understanding of the DDR and differential expression of DDR factors in health and disease, there is promise of not only improving the diagnosing and prognosticating of various cancers but also in guiding treatment decisions and developing new therapies to target these differences between malignant and healthy tissues. This is especially pertinent when it comes to the treatment of cancer since the mechanism of action of non-surgical treatment methods such as radiation therapy (RT) and many types of chemotherapies focus on inflicting DNA damage. Current first-line treatments for OSCC patients with locally advanced, metastatic and recurrent cancers primarily involve single agent platinum-based therapies or in combination with mitotic and/or EGFR inhibitors. Current oral cancer treatment protocols call for chemotherapy with cancer stages III through IVC. These cancers can have tumors graded T1 to T4. Therefore, knowing the different responses to chemotherapy based on tumor grade would be important in determining treatment outcome and, inevitably, treatment success or failure. Although great progress has been made in recent years towards understanding the DDR, there is still a great deal that needs to be investigated to achieve a better understanding of health and disease management.

Though there are hundreds of proteins that play a role in the DDR, there are apical kinases that work together to coordinate the response: Ataxia telangiectasia and Rad3 related protein (ATR), Ataxia telangiectasia mutated (ATM) and DNA-dependent protein kinase (DNA-PKcs) [6–7]. ATR plays a vital role in detecting DNA damage and replication stress as well as initiating checkpoint activation. ATR is also involved as an intermediate of DNA repair, and activates in response to stalled or collapsed replication forks or as a by-product of double stranded breaks (DSBs) [8, 9]. In contrast, ATM and DNA-PKcs are activated in response to DSBs and phosphorylate many of the same substrates, including Replication Protein A (RPA) [10]. RPA, a heterotrimeric protein with subunits of decreasing size termed RPA1, RPA2, and RPA3 is involved in numerous DNA metabolic pathways such as DNA replication, DNA repair, DNA recombination, and the DDR [11, 12]. In response to DNA damage, RPA2 is phosphorylated at multiple sites by ATR, ATM and DNA-PKcs [13–16]. This differential RPA2 phosphorylation has been correlative to predicting resistance to DNA damaging agents in cellular based assays. Manthey et al. identified that a series of OSCC cell lines able to profoundly phosphorylate ATM and DNA-PKcs phosphorylation sites S4 and S8 on RPA2 were more resistant to cisplatin treatment than cells with reduced phosphorylation at S4 and S8 [17]. Their findings suggest that increased S4S8-RPA phosphorylation in response to cisplatin signifies an amplified DDR, halting cell cycle progression and increasing DNA repair, leading to increased drug resistance.

In this study, we determine that immunohistochemical detection of S4S8-RPA phosphorylation can act as a surrogate to determine DDR activation status in human tissues. The resultant amount of phosphorylation is correlative in determining whether a tissue is normal, dysplastic, or cancerous, as well as the grade of tumor. These results suggest that S4S8-RPA phosphorylation may act as a useful clinical indicator of the DDR in oral dysplastic and tumor tissue, and would be clinically beneficial in determining therapeutically efficacious strategies for treatment in stage III–IVC OSCC.

## RESULTS

### RPA is significantly hyperphosphorylated in dysplastic and OSCC tissues

**Figure 1:**
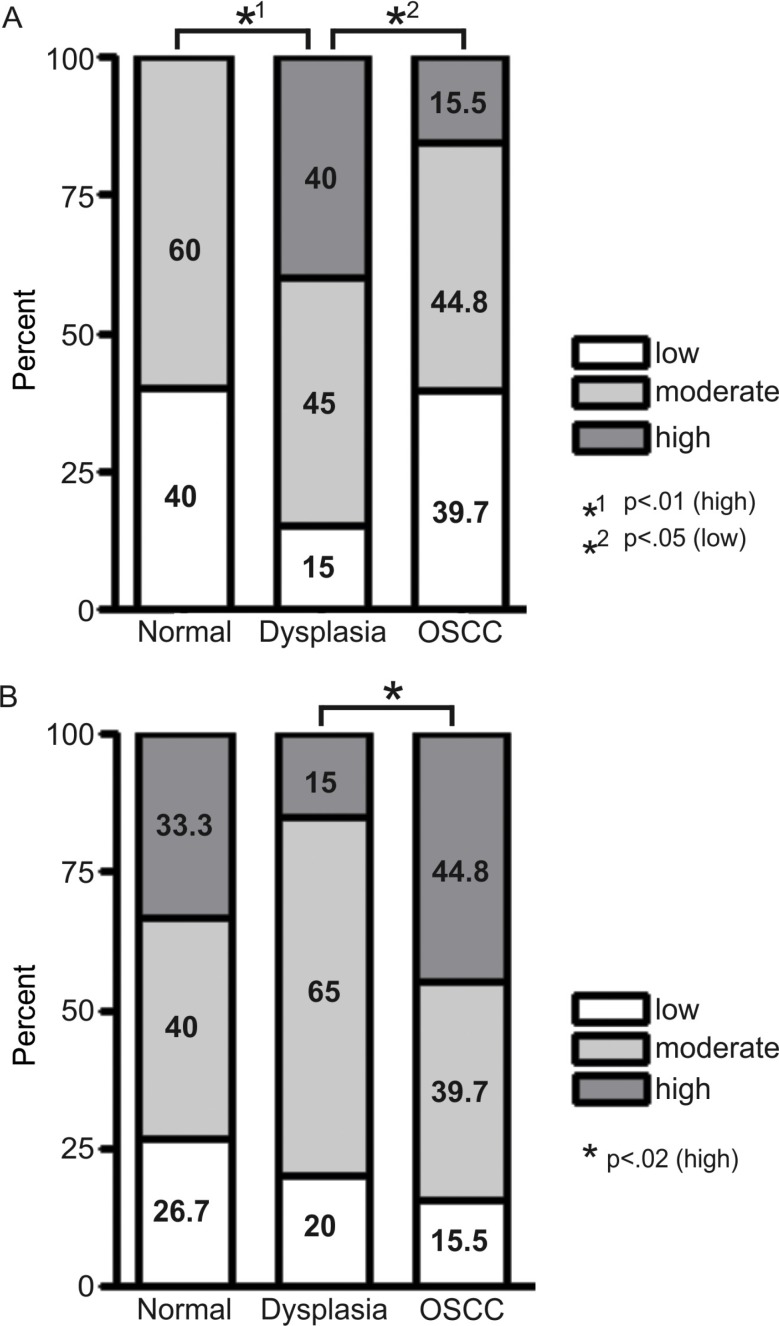
(**A**) Proportional breakdown of S4S8-RPA phosphorylation groups (low, moderate and high phosphorylation) in cells from normal, dysplasia or OSCC tissues. (**B**) Proportional breakdown of overall RPA expression levels (low, moderate and high expression) in cells from normal, dysplasia or OSCC tissues.

Immunohistochemical staining with pS4S8 and RPA antibodies was used to determine S4S8-RPA phosphorylation and RPA expression levels. Samples were grouped according to whether they came from normal, dysplastic or OSCC tissues (Figure 1). In normal tissue (*n* = 15), no samples rated high for S4S8-RPA phosphorylation, while 60% of samples rated as moderate and 40% rated as low (Figure 1A). In dysplasia tissues (*n* = 20), 40% of samples rated high for S4S8-RPA phosphorylation, while 45% were rated as moderate, and 15% rated as low. In OSCC samples (*n* = 58), 15.5% of tissues were rated high for S4S8-RPA phosphorylation, while 44.8% were rated as moderate, and 39.7% rated as low. The high S4S8-RPA phosphorylation proportion difference between normal and dysplasia samples was determined to be highly statistically significant (*p* < 0.01). The low S4S8-RPA phosphorylation proportion difference between dysplasia and OSCC samples was also statistically significant (*p* < 0.05).

For overall RPA expression, normal tissues (*n* = 15) rated 33.3% high, 40% rated moderate, and 26.7% rated low. In dysplasia (*n* = 20), 15% rated high, 65% rated moderate, and 20% rated low. In OSCC (*n* = 58), 44.8% rated high, 39.7% rated moderate, and 15.5% rated low (Figure 1B) All tissue types exhibited similar moderate to high expression of RPA. The high RPA phosphorylation proportion difference between dysplasia and OSCC samples was significant (*p* < 0.02). Representations of low, moderate and high S4S8-RPA phosphorylation and RPA expression are shown in OSCC samples in Supplementary Figure S1. Overall, these initial results suggest that the amount of S4S8-RPA phosphorylation in cells follows the progression of cells from normal to dysplastic and OSCC status.

### S4S8-RPA phosphorylation and RPA expression related to 5-year survival

**Figure 2:**
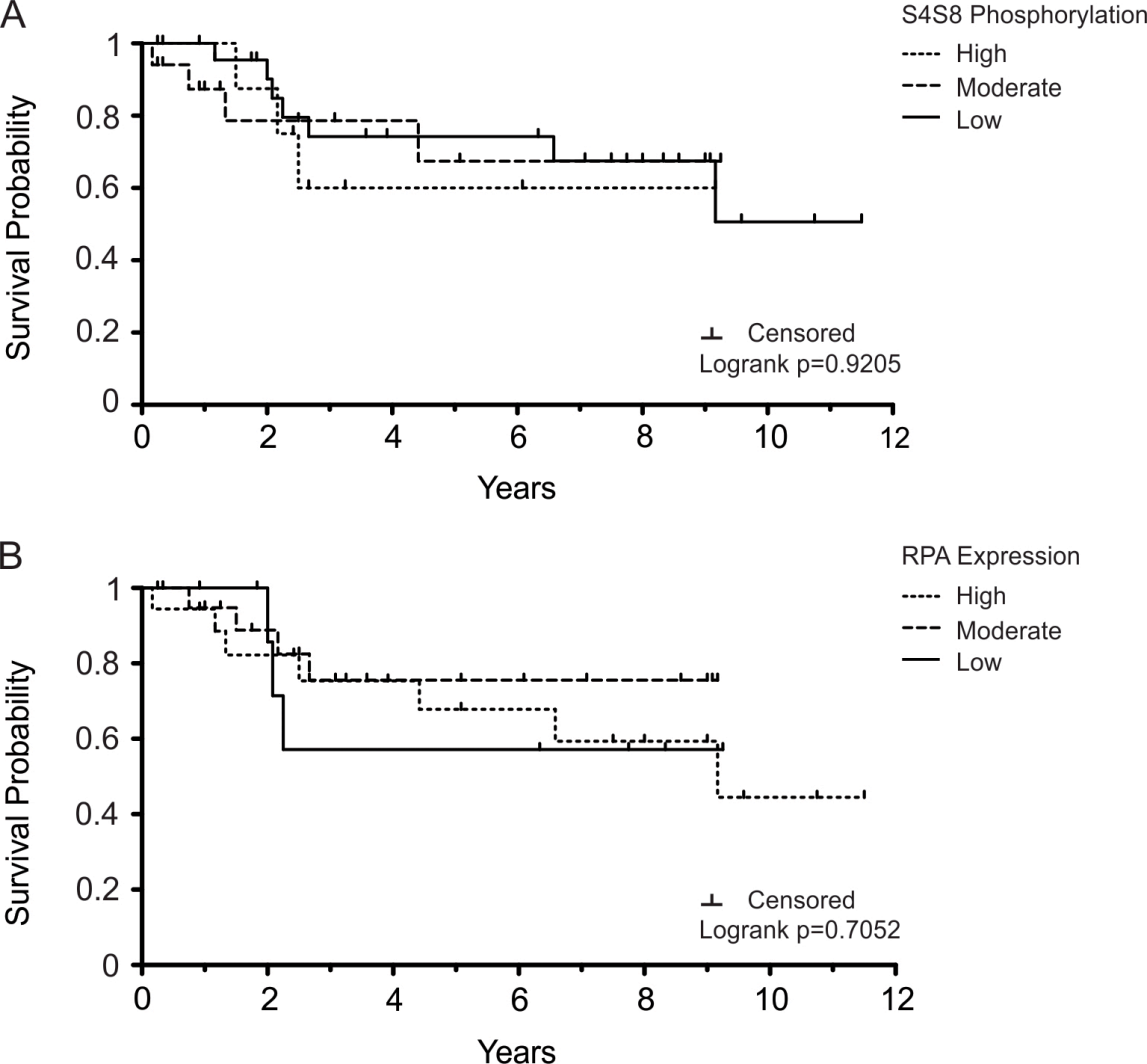
Kaplan-Meier survival plots displaying OSCC survival probability for the patients that were not treated with chemotherapy/radiation therapy Survival probability is displayed in comparison to (**A**) S4S8-RPA phosphorylation levels and (**B**) overall RPA expression levels (low, moderate, high). Censored data is indicated by a tick mark.

The determination that S4S8-RPA phosphorylation greatly increased in dysplastic tissues, then generally decreased in OSCC tissues, is consistent with the model that proposes the DDR acts as a barrier to tumorigenesis in precancerous lesions [18]. Retaining the DDR and p53 function would lead to apoptosis or senescence and possibly decrease viability of the tumor [19, 20]. Therefore, using S4S8-RPA phosphorylation as a surrogate for the DDR, we sought to determine whether the level of S4S8-RPA phosphorylation was possibly linked to OSCC survival probability (Figure 2). The overall 5-year survival rate for our OSCC samples was 68.6% (*n* = 51) (Figure 2A). The 5-year survival probability for high S4S8-RPA phosphorylation group (*n* = 7) was 60%; for the moderate S4S8-RPA phosphorylation group (*n* = 23), 67.41%; for the low S4S8-RPA phosphorylation group (*n* = 21), 74.24%. Survival time was lowest for the high S4S8-RPA phosphorylation group between 2 and 9 years in the study. The difference between the survival probabilities of the groups was not significant (Logrank *p* = 0.9205).

The 5-year survival rate for our high, moderate, and low RPA expression groups were 67.83% (*n* = 25), 75.6% (*n* = 17), and 57.14% (*n* = 9) respectively (Figure 2B). RPA expression had a noticeably different effect on 5-year survival, with low expression tending to result in decreased survival probability. There was no significant difference between the groups (Logrank *p* = 0.7052).

### S4S8-RPA phosphorylation and RPA expression related to 5-year survival within chemotherapy/radiation therapy subgroup

**Figure 3:**
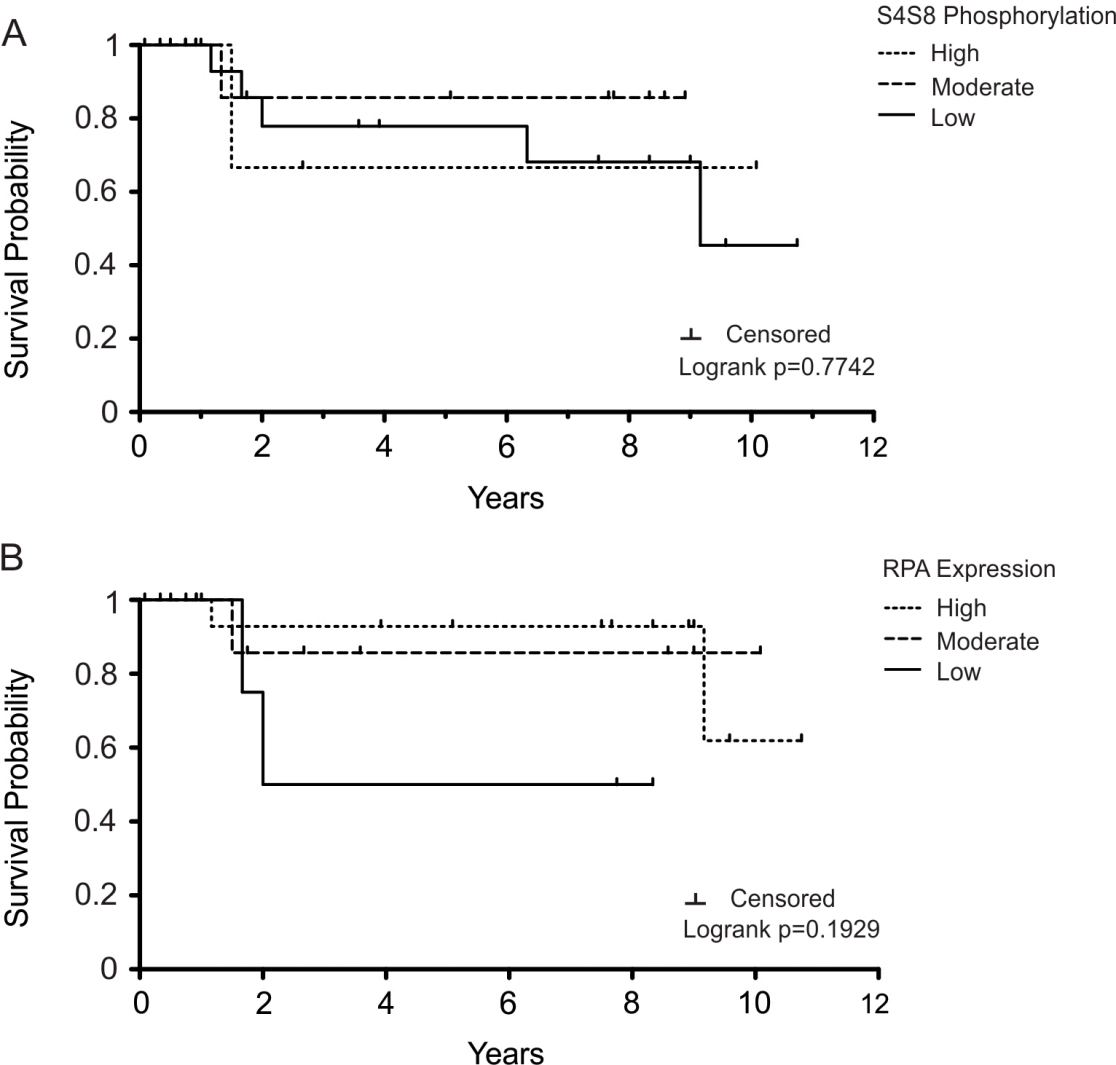
Kaplan-Meier survival plot examining OSCC survival probability in the subgroup of patients who were treated with chemotherapy/radiation therapy Survival probability is displayed in comparison to (**A**) S4S8-RPA phosphorylation and (**B**) overall RPA expression levels (low, moderate, high). Censored data is indicated by a tick mark.

Since cancers with low S4S8-RPA phosphorylation, and therefore low DDR signaling, would likely be more susceptible to therapies that involved DNA damage agents, we evaluated a subgroup of tissues from patients receiving chemotherapy and/or radiation therapy in addition to surgery (*n* = 29) (Figure 3). The 5-year survival probabilities of the high, moderate, and low S4S8-RPA phosphorylation groups were 66.67% (*n* = 3), 85.71% (*n* = 15) and 77.92% (*n* = 11) respectively (Figure 3A). Due to the small number of samples, the differences within this grouping are not significant (Logrank *p* = 0.7742). However, this result trends to the possibility that high S4S8-RPA phosphorylation in OSCC leads to poorer prognosis possibly due to resistance to DNA damaging agents.

The 5-year survival probabilities of high, moderate, and low RPA-expression groups within the chemotherapy/radiation subgroup were 92.86% (*n* = 17), 85.74% (*n* = 8), and 50% (*n* = 4) respectively (Figure 3B). There was no significant difference between the groups (Logrank *p* = 0.1929).

### Relative S4S8 phosphorylation is an indicator of tumor progression

**Figure 4:**
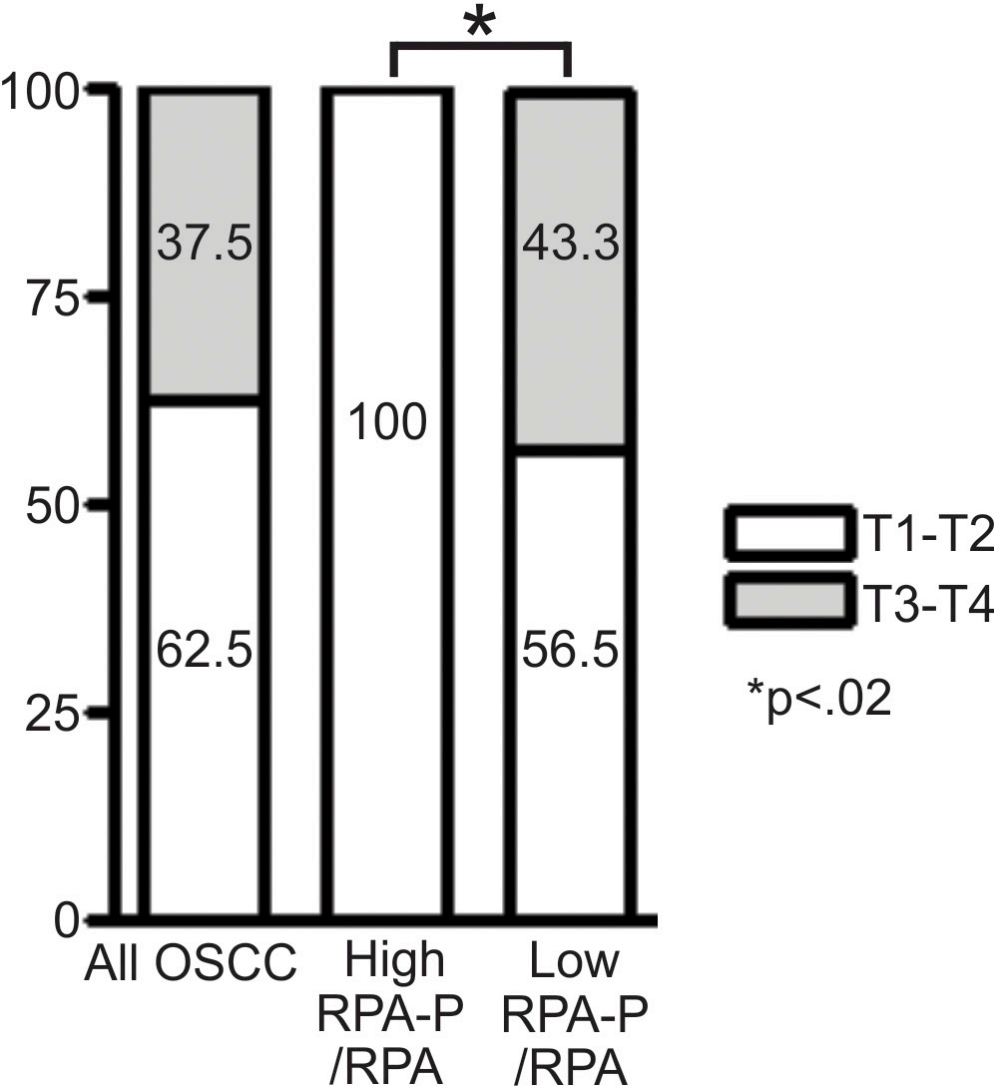
Chart displaying the proportional breakdown of OSCC tumor grade classification (T1-T2, T3-T4) in comparison to relative low or high S4S8-RPA phosphorylation levels (All OSCC (*n* = 56); High RPA-P (*n* = 8); Low RPA-P (*n* = 30)

To further assess 5-year survival rate and RPA hyperphosphorylation status, we created subgroups of relative-high hyperphosphorylating tissues (having S4S8-RPA phosphorylation amounts high relative to RPA expression), and relative-low hyperphosphorylating tissues (having S4S8-RPA phosphorylation amounts low relative to RPA expression). We examined the relationship between relative-high S4S8-RPA phosphorylation and relative-low S4S8-RPA phosphorylation in relation to tumor stages, T1-T2 and T3-T4 (Figure 4) and in comparison to the entire OSCC sample. In this comparison, none of the relative-high S4S8-RPA phosphorylation OSCC samples were graded to T3-T4 stages while the relative-low S4S8-RPA phosphorylation samples revealed higher graded T3-T4 stage tumors in 43.3% of the samples. The significant difference (*p* = 0.022) between the relative low and high S4S8-RPA phosphorylation group tumor stage grouping indicates that relative S4S8-RPA phosphorylation is higher in early stage tumor progression.

## DISCUSSION

A recently developed model for cancer progression argues that oncogene-induced DNA damage leads to activation of pathways, including S4S8-RPA phosphorylation, that serve as a barrier to cancer progression [21]. Our finding of a significant increase in S4S8-RPA phosphorylation observed in dysplasia compared to normal and OSCC tissues support this model. This finding is consistent with other studies which demonstrate an increased DDR in precancerous tissues compared to normal tissue, followed by a drop in DDR in mature tumors [18, 21]. Further, if we interpret RPA expression as a marker for replication, the potential decrease in RPA expression noted in precancerous tissues is consistent with results from studies of colon cancer progression [21]. For example, Bartkova et al, using IHC methods, demonstrated an increased activation of the ATM-Chk2-p53 checkpoint pathway in early, superficial urinary bladder cancer lesions compared to normal and advanced tumors [18]. Considering ATM is one of two primary kinases that phosphorylate RPA2 at S4 and S8, it is reasonable to suggest that RPA phosphorylation also plays a role in this tumorigenesis barrier.

In the examination of the relative low and high S4S8-RPA phosphorylation groups to tumor stage, all 8 of the high S4S8-RPA phosphorylation tumors in this study were early stage, T1-T2 tumors, compared to the low S4S8-RPA phosphorylation group which had close to half of the samples as late stage (T3-T4) tumors. This pattern of S4S8-RPA phosphorylation is consistent with previous studies which demonstrated down-regulation of DDR expression in advanced-stage tumors versus early stage tumors [18]. The presence of high S4S8-RPA phosphorylation, and therefore intact DDR signaling, in dysplasia and T1-T2 graded tumors, suggests that these cells would likely be resistant to first-line platinum-based chemotherapies and would require higher doses or alternate treatments such as cetuximab that target signaling pathways other than DDR signaling. Late stage T3-T4 tumors with inactive DDR are less likely to have G1 and G2 checkpoints due to loss of p53 and apical kinase functions involved in RPA phosphorylation which would suggest a higher susceptibility and selectivity with platinum-based chemotherapies. Indeed, cells expressing S4S8 phosphorylation mutants that are unable to phosphorylate Ser4 and Ser8 are defective in G2/M arrest and experience increased mitotic catastrophe in response to chemotherapeutics [14, 22]. This is consistent with our previous findings that correlate the inability of OSCC cells to phosphorylate S4S8-RPA in response to DNA damaging agents with increased sensitivity to these chemotherapeutics [17].

In this study, we identified increased S4S8-RPA phosphorylation in dysplastic tissues which may indicate the activation of a barrier to tumor progression. In addition, we provide evidence of down-regulation of the DDR in advanced-stage tumors compared to early-stage tumors using S4S8-RPA phosphorylation. Our data suggest analyzing levels of S4S8-RPA phosphorylation in untreated tumors may have limited advantages as compared to analyzing levels in tumors exposed to chemotherapy. Results of S4S8-RPA phosphorylation did not predict survival in the untreated tumor groups; however, trends observed in 5-year survival rates of those receiving chemo- and radiotherapy suggest promise of S4S8-RPA phosphorylation analysis as potentially useful in further chemotherapy treatment choices. This initial study demonstrates promising results and merits larger scaled studies to examine the role of S4S8-RPA phosphorylation in OSCC in the future.

## MATERIALS AND METHODS

### Antibodies

Primary antibodies used for Immunohistochemistry (IHC) are mouse anti-RPA2 (9H8, Santa Cruz Biotechnology, Dallas, TX) and rabbit anti-S4S8 Phospho RPA32 (Bethyl Laboratories Inc, Montgomery, TX). Secondary antibodies and IHC for detection of RPA32 was carried out using Anti-mouse Ig HRP Detection Kit (BD Biosciences, San Jose, CA). For pS4S8-RPA2 detection, anti-rabbit Bethyl IHC Accessory Kit was used.

### Tissue samples used for immunohistochemistry

Normal (non-dysplastic or cancerous) and dysplasia human biological material (HBM) tissues used for immunohistochemistry were obtained from archived paraffin-embedded, formalin-fixed tissue samples from the University of Nebraska Medical Center College of Dentistry Oral Pathology biopsy service in Lincoln, NE. Oral, laryngeal, and hypopharyngeal squamous cell carcinoma (OSCC) tissues were obtained in the form of paraffin-embedded slides from the University of Nebraska Medical Center College of Medicine Department of Pathology and Microbiology in Omaha, NE. When applicable, biographical data and disease-specific medical history was obtained from the UNMC College of Medicine Department of Otolaryngology/Head & Neck Surgery in Omaha, NE. Institutional Review Board (IRB) approval was obtained for the use of collected HBM and relevant medical data. 15 normal, 20 dysplasia, and 58 OSCC tissues were examined in this study.

### Immunohistochemistry

From paraffin-embedded tissue blocks, 4–6 μm thick sections were prepared on slides for IHC. Negative control slides were processed without primary antibody. High-staining tissues were used as positive controls. Slides were deparaffinized and rehydrated with three stages of xylene immersion (5′) and decreasing concentrations of ethanol from 100%(2 × 5′), 95% (2 × 3′), 80% (3′), 70% (3′), 50% (3′). Slides were then treated for 10 minutes with 3% hydrogen peroxide (H_2_O_2_) to block endogenous peroxidase activity. Antigen retrieval was performed by a 20 minute water bath in Tris EDTA buffer (pH 9.0) between 96–99 degrees Celsius. Slides were allowed to cool at room temperature for 30 minutes. Slides were washed with IHC solution wash (Bethyl). Primary antibody incubation was performed at 1:100–125 dilution overnight in a condition-controlled cold room. After washing slides, biotinylated anti-mouse secondary antibody was incubated at 1:100 dilution and according to manufacturer's recommendations for the pre-diluted anti-rabbit Ab for one hour at room temperature. Following slide washing, RPA2 antibody-treated slides were treated with streptavidin-HRP for 30 minutes and washed. S4S8-RPA antibody treated slides had HRP conjugated directly to secondary antibody. Diaminobenzene (DAB) exposure for 2–5 minutes was then followed by distilled water wash (3 × 3′). Slides were counterstained with Meyer's hematoxylin for 2 minutes, and washed in a basic bluing solution or running tap water. Slides were then dehydrated with increasing concentrations of ethanol (95% 2 × 5′, 100% 2 × 5′) and xylene immersion (3 × 5′) to prepare for slide mounting.

### Evaluation of immunohistochemistry tissue staining

Slides were independently evaluated by two calibrated examiners and rated as high, moderate, or low expression based on the relative number of cells stained per section as well as the intensity of staining (Supplementary Figure S1). A third calibrated examiner was consulted in the event that the first two examiners did not agree on the staining classification. In cases where a third calibrated examiner was consulted and a majority was not achieved (high, moderate, and low grades), slide ratings were averaged and rated as moderate.

### Evaluation of treatment outcome

Relevant treatment outcome history was obtained for OSCC tissue donors when applicable, including TNM stage, histological grade, 5-year survival probability, and tumor recurrence. 5-year survival was determined for all possible OSCC tissues. Tumor TNM stage and histological grade were determined by a certified pathologist.

### Statistical analysis

Chi-squared tests for independence was performed to evaluate differences in S4S8-RPA phosphorylation proportions and RPA expression proportions between groups. Statistical significance was considered at a *p-value* < 0.05. Logrank scores were computed to test for the difference in survival probabilities between high, moderate, and low S4S8-RPA phosphorylation as well as RPA expression. Survival probability statistics and Kaplan-Meier curves were generated with GraphPad Prism 4.

## SUPPLEMENTARY MATERIALS FIGURE


