# Supplementary material for: S4S8-RPA phosphorylation as an indicator of cancer progression in oral squamous cell carcinomas
Source: Oncotarget. 2016 Dec 16;8(6):9243–50. doi: 10.18632/oncotarget.14001 (PMC5354728; doi:10.18632/oncotarget.14001)
Supplement: Supplementary file 1 [file oncotarget-08-9243-s001.pdf]

S4S8-RPA phosphorylation as an indicator of cancer progression in oral squamous cell carcinomas

Supplementary Materials

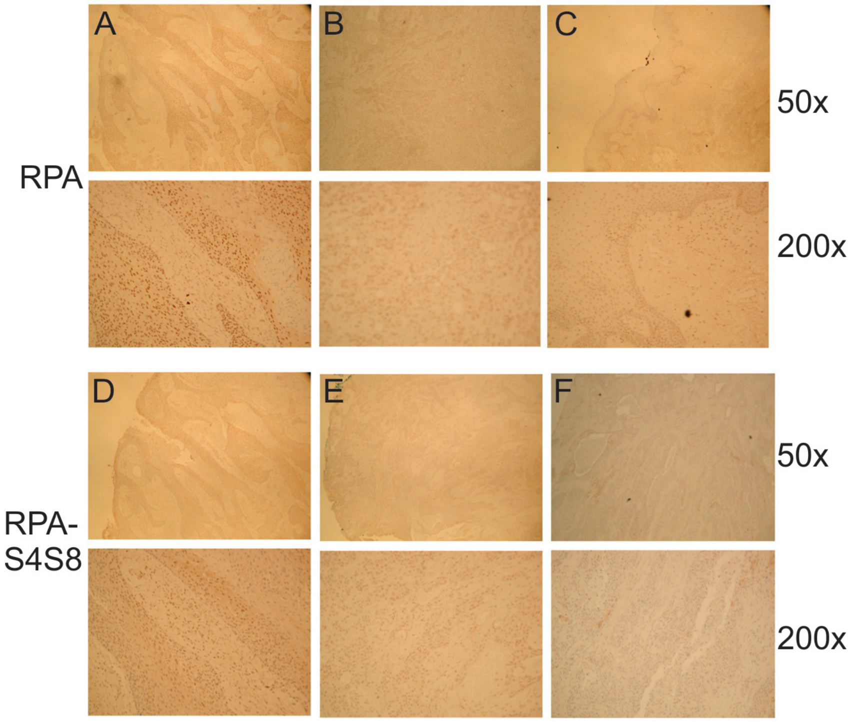

Supplementary Figure S1: Representative images of RPA (**A, B, C**) and S4S8-RPA (**D, E, F**) staining observed in OSCC via immunohistochemistry. Examples of representative of high (A, D), medium (B, E) and low (C, F) antibody signal at 50X and 200X.
